# Association between vigorous physical activity and life satisfaction in adolescents

**DOI:** 10.3389/fpubh.2022.944620

**Published:** 2022-10-13

**Authors:** Bin Feng, Ke Xu, Panpan Zhou

**Affiliations:** ^1^Department of Physical Education, Tianjin College, University of Science and Technology Beijing, Tianjin, China; ^2^HBU-UCLan School of Media Communication and Creative Industries, Hebei University, Baoding, China

**Keywords:** vigorous physical activity, adolescence, life satisfaction, health, HSBC

## Abstract

**Purpose:**

The association between overall physical activity (PA) and life satisfaction has been confirmed in adolescents. However, the associations between different forms of PA at various intensities and life satisfaction are under-studied. This study aimed to explore the association between vigorous PA (VPA) and life satisfaction, and whether the associations vary by gender and age.

**Methods:**

Using data from the Health Behavior in School-aged Children (HSBC) investigation, the research samples of 11- to 15-year-old adolescents were included for further. The information on VPA was collected *via* two self-reported questions, including frequency per week and hours per week. Life satisfaction was assessed by a ladder of 0–10 scores, with being higher indicating better life satisfaction. Logistic models were carried out to analyse the association between VPA and life satisfaction among adolescents, and results were presented with odd ratio (OR) and associated 95% confidence interval.

**Results:**

Among the 214,080 (49.2% male) adolescents studied, both boy and girl participants reporting higher frequency of VPA per week were more likely to be associated with higher life satisfaction (e.g., in boys, VPA for every day: OR = 1.054; in girls: VPA for every day: OR = 1.047). More hours of VPA was also associated with better life satisfaction in adolescents (in overall sample, 7 h or more: OR = 1.178).

**Conclusions:**

In conclusion, this research provided evidence on the roles of VPA on life satisfaction among adolescents aged 11–15. Considering life satisfaction is regarded as an indicator of adolescents' psychological health development, our study supports and extends the evidence for the importance of VPA in adolescence.

## Introduction

Mental health educators and professionals are concerned about adolescents' well-being promotion (1–3). A lot of constructs related well-being are proposed, and it is widely recognized that fostering an understanding of life satisfaction is important to goal achievement (4, 5). Life satisfaction is an overall and cognitive appraisal of individuals' life (6, 7). Life satisfaction is a crucial component of positive psychology, which can be defined as a subjective sensitivity of the multiple life aspects in people (8–10). Life satisfaction involves a cognitive assessment in humans' whole character of life (11), which also includes self-satisfaction, family satisfaction, as well as school satisfaction (12). Better life satisfaction is linked with improved health outcomes (13). Besides, life satisfaction has also been regarded as being related to future externalizing, internalizing behaviors and peer victimization experiences in previous studies (14, 15). Life satisfaction is also a predictor of health-related quality of life (16), behavioral problems (17) (i.e., violence (18)) and psychological problems (i.e., depressive disorders (19), suicidal ideation (20)) in adolescents. Empirical evidence indicated that a decline in life satisfaction in adolescents (21–24).

Owing to the significance and practical implications, improving life satisfaction is of great importance. According to the empirical evidence, physical activity (PA) has been regarded as an optative strategy to boost children's and adolescents' mental (25) and physical health (26, 27). Recently, there is evidence showing that higher PA levels are related to children's and adolescents' life satisfaction (10, 28). In general, PA and life satisfaction's positive relationship has been reported in several previous studies (29–32). For example, PA was positively related with life satisfaction in US middle school children (29). Besides, one cross-sectional research that investigated 4,758 adolescents in South Carolina revealed that life satisfaction was favorably associated with PA, and gender and ethnicity had effect on this association (30). Another study of 1,002 Chinese middle school students also supported the positive association, and adolescents who participated in more PA had a higher life satisfaction (31). Besides, results from the CASPIAN-IV study demonstrated that PA may be a better way to achieve optimal life satisfaction than reducing screen time among children and adolescents (33, 34).

Although the associations between overall PA and life satisfaction have been established, some research gaps remain, that needs to be addressed by future research. The first one is that little is known about the different forms of PA and life satisfaction. As we implied above, one evident research gap is rare evidence concerning VPA and life satisfaction. If researchers can verify the association between VPA and life satisfaction in adolescents, it would be more beneficial to promote wellbeing in this population subgroup. Second, previous studies mostly focused on adolescents from single country. This lack of evidence from multiple countries would limit research findings generalization, as evidence based on more countries or regions can be applied widely for adolescents' health promotion. For example, one recent study highlighted a positive association between frequency of VPA and life satisfaction Italian Adolescents (35). Unclearly, it cannot be judged whether VPA or specific PAs have a positive influence on life satisfaction in other countries.

Thus, this study aims to explore the associations between VPA and life satisfaction in adolescents using a multiple-country data. This study hypotheses that high participationsof VPA would be associated with higher life satisfaction in adolescents.

## Methods

### Study design and participants

With the public data from the Health Behavior School-aged Children (HBSC) survey, we conducted this cross-sectional research and studied it in accordance with the international HBSC protocol. HBSC is considered a multinational collaborative WHO research that gathers health behavior data every 4 years from a nationally typical sample of 11–13–15 ages' adolescents, where “school classes” will be considered the main sampling unit. Systematic bunch sampling applies the population's eventuality proportional to obtain a nationally typical sample. The samples' class teachers were told and asked to transmit each student an information sheet that included some basic information about the research, which was expected to be provided to their parents or guardians (27). An opt-out form will be signed and returned only in the event of refusal to participate. Study participants were invited to take in this survey voluntarily. A formal consent from study participants was obtained prior to data collection.

Data required were collected in an anonymous manner and standard safeguards to ensure confidentiality. A well-trained teacher granted a normal and self-made questionnaire to students. According to the survey in 2013/2014, the reaction rate of school/class and student level in most areas exceeded 85%.

### Measures

#### Vigorous physical activity (independent variables)

VPA was surveyed by the two terms in below. The first item was “In your spare time, how many opportunities do you have to exercise until you're out of breath or sweaty?” The answers maybe *every day, 4–6 times/week, 2–3 times/week, once a week, once a month even less, and never*. The second item was “Outside school hours, how many hours per week do you typically exercise during spare time to get breathless or sweaty? “The answers maybe *zero*, ~*0.5 h*, ~*1 h, about 2–3 h, about 4–6 h, about 7 h even more*.

#### Life satisfaction (outcome)

Life satisfaction was assessed based on the ladder life scale (36). The participants were asked to tick the box. The ladder numbers correspond to 0–10 from bottom to top, with 10 at the top representing the best life, and 0 at the bottom representing the worst life. Which class do you think you are in now? 10 is the best life, 0 is the worst, previous research has shown that even a single life satisfaction survey can be very accurate, very valid, credible. The scores are also high and are basically the same as multiple measures of life satisfaction. (37–39).

#### Covariates

Sociodemographic factors included sex (boy or girl), age (11, 13 and 15 years), alcohol use last 30 days (On how many days have you drunk alcohol?), socioeconomic status (family well off), smoking (in the last 30 days, on how many days have you smoked cigarettes?), watching television/, playing computer games and body mass index (BMI). Family well off was assessed by a question, that was “How well of do you think your family is?”. BMI was estimated by self-reported height and weight. The reason of above covariates were chosen is that they were included in previous studies on physical activity and mental health in children and adolescents (40). According to the previous studies, these controlling variables (e.g., drinking alcohol, family well off, smoking, watching tv/DVD/video, playing computer games, vigorous physical activity and BMI) were highly linked with life satisfaction (41–44). In addition, socioeconomic status is also associated with life satisfaction (35).

### Statistical process

All the statistical analysis was performed using the SPSS 25.0. Prior to final analysis, some procedures have been made. Study participants provided valid cases of variables of interest of this study were targeted for further analysis. Missing cases of variables of interest were deal with completed case analysis, as the proportion of missing case was under 8% of total sample. Descriptive analysis was used to report sample characteristics. Categorical data are shown as numbers and percentages, while continuous data are displayed as the mean and standard deviation. The Kolmogorov–Smirnov test and normal probability plot were applied to test the assumption of normality of continuous variables (i.e., BMI and life satisfaction). To assess the associations between VPA and life satisfaction, Generalized Linear Models were used as this model is highly compatible with the different kinds of distributions of the studied variables. Three models were established, the first one was the associations between VPA and life satisfaction in the overall sample while controlling for all the covariates. The second model concerned with the associations between VPA and life satisfaction by sex while controlling for all the covariates except for sex. The third model concerned with the association between VPA and life satisfaction by age while controlling for all the covariates except for age. In all the models, “*none*” (hours of VPA per week) and “*never*” (frequency of VPA per week) were treated as reference group. Odd ratio (OR) with 95% confidence interval (CI) was presented. The statistical significance was set up as *p* < 0.05.

## Results

Supplementary Table S1 presents several characteristics of the sample adopted in this study. The sample consisted of 105,414 boys and 108,666 girls, accounting for 49.2 and 50.8% of the total sample, respectively. Besides, the largest proportion of adolescents in the entire sample, 34.6%, were 13 years old, while adolescents aged 11 and 15 years old occupied 32.2 and 33.1% separately, in the study. In addition, among all adolescents those who never smoked or had not consumed alcohol in the past 30 days made up most of the sample. However, they generally spent half an hour per day playing computer games on weekdays but spent 2 h or less a day on weekends basically. Finally, the average body mass index (BMI) of adolescents reached 19.6, and their life satisfaction level on average was about 8. Information on study participants by country can be found in Supplementary Table S2.

As shown in Table 1, for the whole sample, the highest likelihood of reporting high life satisfaction was observed among participants who exercised more than once a week (Every day: OR = 1.050; 95% CI: 1.041–1.059; 4-6 times a week: OR = 1.025; 95% CI: 1.016–1.034; 2-3 times a week: OR = 1.016; 95% CI: 1.007, 1.024). On the contrary, in general, the possibilities of reporting better life satisfaction were significantly higher in the participants with more hours spent for VPA.

**Table 1:** Association between vigorous physical activity and life satisfaction.

**Overall**				
**Frequency**	***p***	**OR**	**95%CI**	
Every day	0.000	**1.050**	**1.041**	**1.059**
4-6 times a week	0.000	**1.025**	**1.016**	**1.034**
2-3 times a week	0.000	**1.016**	**1.007**	**1.024**
Once a week	0.767	1.001	0.993	1.010
Once a month	0.984	1.000	0.990	1.010
Less than once a month	0.001	0.983	0.974	0.993
Never	REF
**Hours a week**	***p***	**OR**	**95%CI**	
None	REF
Half an hour	0.003	**1.070**	**1.023**	**1.119**
1 hour	0.000	**1.098**	**1.050**	**1.148**
2-3 hours	0.000	**1.144**	**1.094**	**1.197**
4-6 hours	0.000	**1.145**	**1.091**	**1.202**
7 hours or more	0.000	**1.178**	**1.120**	**1.239**

OR, odd ratio; CI, confidence interval; REF, reference group.

Bold fonts denote statistical significance.

Models controlled for sex, age, family socioeconomic status, smoking, alcohol use, television viewing time, computer games time, computer use time, body mass index.

Moreover, Table 2 illustrates the relationship between VPA and life satisfaction of the participants by gender. VPA was related to higher life satisfaction in both boys (OR = 1.054; 95% CI: 1.041, 1.067), and girls (OR = 1.047; 95% CI: 1.034, 1.059). Similarly, VPA more than twice a week was associated with higher life satisfaction in both boys and girls as well. Meanwhile, life satisfaction was reported to be higher as the hours of VPA a week increased.

**Table 2:** Association between vigorous physical activity and life satisfaction by gender.

**Boy**				
**Frequency**	***p***	**OR**	**95%CI**	
Every day	0.000	**1.054**	**1.041**	**1.067**
4–6 times a week	0.000	**1.031**	**1.019**	**1.044**
2–3 times a week	0.000	**1.014**	**1.002**	**1.026**
Once a week	0.07	1.002	0.990	1.015
Once a month	0.07	0.995	0.980	1.011
Less than once a month	0.08	0.992	0.977	1.006
Never	REF
**Hours a week**	***p***	**OR**	**95%CI**	
None	REF
Half an hour	0.770	1.010	0.944	1.081
1 h	0.036	**1.073**	**1.005**	**1.146**
2–3 h	0.000	**1.154**	**1.082**	**1.231**
4–6 h	0.000	**1.147**	**1.071**	**1.228**
7 h or more	0.000	**1.188**	**1.108**	**1.273**
**Girl**
**Frequency**	***p***	**OR**	**95%CI**	
Every day	0.000	**1.047**	**1.034**	**1.059**
4–6 times a week	0.000	**1.018**	**1.006**	**1.030**
2–3 times a week	0.000	**1.017**	**1.005**	**1.029**
Once a week	0.10	1.000	0.988	1.012
Once a month	0.07	1.003	0.989	1.017
Less than once a month	0.06	0.981	0.968	0.993
Never	REF
**Hours a week**	***p***	**OR**	**95%CI**	
None	Ref
Half an hour	0.001	**1.106**	**1.042**	**1.175**
1 h	0.000	**1.118**	**1.052**	**1.187**
2–3 h	0.000	**1.130**	**1.062**	**1.202**
4–6 h	0.000	**1.141**	**1.066**	**1.222**
7 h or more	0.001	**1.333**	**1.051**	**1.221**

OR, odd ratio; CI, confidence interval; REF, reference group.

Bold fonts denote statistical significance.

Models controlled for age, family socioeconomic status, smoking, alcohol use, television viewing time, computer games time, computer use time, body mass index.

Results for the relationship between VPA and life satisfaction by age are illustrated in Table 3. In general, adolescents aged 13 and 15 years old who had VPA more frequently per week appeared more likely to report high life satisfaction. In terms of VPA hours per week, adolescents aged 13 or 15 years who engaged in more tended to report higher life satisfaction. Conversely, adolescents aged 11 years, especially for those engaging in VPA every day (OR = 1.335; 95% CI: 1.200–1.485) or those with more than 7 h a week for VPA (OR = 1.222; 95% CI: 1.016–1.238) were more likely to report higher life satisfaction.

**Table 3:** Association between vigorous physical activity and life satisfaction by age group.

**11 years**				
**Frequency**	***p***	**OR**	**95%CI**	
Every day	0.000	**1.335**	**1.200**	**1.485**
4–6 times a week	0.023	1.132	1.017	1.26
2–3 times a week	0.593	1.029	0.927	1.143
Once a week	0.098	0.91	0.814	1.017
Once a month	0.300	0.926	0.8	1.071
Less than once a month	0.021	0.857	0.752	0.977
Never	REF
**Hours a week**	***p***	**OR**	**95%CI**	
None	REF
Half an hour	0.943	0.997	0.914	1.087
1 h	0.052	1.088	0.999	1.185
2–3 h	0.087	1.078	0.989	1.174
4–6 h	0.259	1.055	0.961	1.158
7 h or more	0.023	**1.122**	**1.016**	**1.238**
**13 years**
**Frequency**	***p***	**OR**	**95%CI**	
Every day	0.000	**1.600**	**1.447**	**1.769**
4–6 times a week	0.000	**1.305**	**1.182**	**1.442**
2–3 times a week	0.000	**1.216**	**1.103**	**1.341**
Once a week	0.256	1.06	0.959	1.172
Once a month	0.662	1.027	0.911	1.158
Less than once a month	0.806	0.986	0.882	1.103
Never	REF
**Hours a week**	***p***	**OR**	**95%CI**	
None	Ref
Half an hour	0.006	**1.119**	**1.033**	**1.211**
1 h	0.001	**1.143**	**1.057**	**1.236**
2–3 h	0.000	**1.223**	**1.131**	**1.323**
4–6 h	0.000	**1.244**	**1.144**	**1.354**
7 h or more	0.000	**1.316**	**1.206**	**1.437**
**15 years**
**Frequency**	***p***	**OR**	**95%CI**	
Every day	0.000	**1.758**	**1.605**	**1.927**
4–6 times a week	0.000	**1.426**	**1.301**	**1.562**
2–3 times a week	0.000	**1.261**	**1.153**	**1.379**
Once a week	0.000	**1.181**	**1.077**	**1.295**
Once a month	0.004	**1.166**	**1.051**	**1.295**
Less than once a month	0.495	0.967	0.878	1.065
Never	REF
**Hours a week**	***p***	**OR**	**95%CI**	
None	Ref
Half an hour	0.131	1.056	0.984	1.135
1 h	0.228	1.044	0.973	1.121
2–3 h	0.000	**1.153**	**1.074**	**1.238**
4–6 h	0.000	**1.166**	**1.08**	**1.259**
7 h or more	0.000	**1.168**	**1.079**	**1.265**

OR, odd ratio; CI, confidence interval; REF, reference group.

Bold fonts denote statistical significance.

Models controlled for sex, family socioeconomic status, smoking, alcohol use, television viewing time, computer games time, computer use time, body mass index.

## Discussion

This study aimed to explore the associations between frequency and duration of VPA and life satisfaction in adolescents aged 11–15 years. The results generally suggested a relationship between a higher frequency of VPA and higher levels of life satisfaction in adolescents regardless of gender differences. Farren et al. (45) discovered that VPA presented a negative relationship with depression symptoms in adolescents. The authors, based on their results, recommended indirectly improving the quality of life and mental health of adolescents by increasing VPA. In addition, Hrafnkelsdottir et al. found that adolescents in Iceland who undertook more frequent VPA together with decreased screen time were less likely to report mental health problems. One research carried out by Bélair et al. (46) concluded that low levels of VPA were related to depression symptoms, based on the evidence from the sample of Canadian adolescents. This indicated that more frequent VPA should contribute to lower depression levels in adolescents, resulting in better life satisfaction. Additionally, the study carried out by Costigan et al. (47) further emphasized the role played by frequent VPA in improving life satisfaction in adolescents, recommending VPA for adolescents three times a week. For this regard, Oberste et al. (48) suggested that MPA or VPA played an essential role in treating mental health in adolescents. One of the most prevalent explanations for this finding is that PA prevents underlying mental health problems such as depression, anxiety, lower self-esteem, and suicidal tendencies, therefore increasing life satisfaction levels in youth (49).

Adolescents with more hours spent in VPA outside of school hours were less likely to report higher scores of life satisfaction, especially in those with none, half an hour and 1 h a week for VPA. These findings are consistent with the positive associations between VPA and adolescents' better life satisfaction in several previous studies (50–52). The positive association may be interpreted by Graham et al. (1) increased physical health (i.e., Cardiometabolic fitness (53), functional capacity (54)). Besides, another explanation might be the effects of physical activity on psychological problems, such as anxiety, depressive symptoms (55). These benefits that adolescents obtain from physical activity might increase their satisfaction with life. Data from one HBSC study revealed that VPA was a stronger predictor of life satisfaction than MVPA in adolescents, especially for boys, which implied that the promotion of VPA might be an efficient way to foster life satisfaction among adolescents (51). Thus, not participating in PA not only has an impact on current life satisfaction but also is associated with dissatisfaction during adulthood (30). Although socioeconomic status was not the aim of this study, it has been regarded as a confounding factor.

As implied by the results, there was a age difference in the association between VPA and life satisfaction. This finding is consistent with the results presented by GarciaGarcia et al. (56) that VPA of children and adolescents aged 11 did not play a significant role in the health-related quality of life (HRQoL) and less maturity groups' correlation. Other researchers have reached different outcomes which mainly on the VPA duration and life satisfaction's correlation among adolescents' subgroups by age. On one hand, Olofsson et al. (57) suggested a negative association between VPA and HRQoL as well as life satisfaction in adolescents. Nevertheless, it should be noted that the adolescents participating in the study were treated for isolated congenital valvular aortic stenosis, which means that the results achieved by the scholars might be distinct among healthy adolescents. On the other hand, other researchers have discovered a positive connection between the duration of VPA and life satisfaction levels among adolescents. According to Cao et al. (58), the study carried out among teenagers aged 11–16 indicated that frequent VPA of sufficient time should be a protective factor for depression among the participants including boys and girls from junior high schools, which means that sufficient VPA was positively associated with more life satisfaction among adolescents. Besides, although Urchaga et al. (59) did not specify the connection between VPA and life satisfaction for the 12–16-year-old adolescents in the sample, they recommended in their study that school-age adolescents should conduct VPA for 60 min at a time.

### Study strengths and limitations

The main strengths are standardized and validated data collection procedures, based on the international HBSC study, and the use of a large and representative sample to investigate the association between VPA and life satisfaction in adolescents. Some inherent limitations should be acknowledged within this study's participants, measure and design. First, because the design of the study is cross-sectional, no causal conclusion could be drawn from the current study; it is impossible to make sure the orientation of independence and outcomes' connection. Second, the self-reported measures are taken in the study to collect variable data, and they are subject to the social desirability of participants and the recall bias. Due to the limitations, it is suggested that future research focus on the generation of strong proof. Although there are some mentioned restrictions, but the research also has advantages.

## Conclusion

In conclusion, this cross-sectional study suggested that more frequent VPA might associated with better life satisfaction among adolescents. however, this association between may be influence by age of adolescents and this research finding needs further confirmation. Generally, with the purpose of achieving higher life satisfaction among adolescents, VPA with higher frequency and longer duration is recommended. The practical implication of this study is to encourage the future research may be paid specific attention on the association VPA related to life satisfaction, which would be conducive to well-being promotion in adolescents.

## Data availability statement

The original contributions presented in the study are included in the article/Supplementary materials, further inquiries can be directed to the corresponding authors.

## Ethics statement

All data were anonymized and publicly available; therefore no ethical approval was required. Written informed consent to participate in this study was provided by the participants' legal guardian/next of kin.

## Author contributions

BF: writing—original draft. KX and PZ: formal analysis. BF, KX, and PZ: writing—review and editing. All authors contributed to the article and approved the submitted version.

## Funding

BF was funded by Hebei Social Sciences Foundation Project: Empirical Research on the Path of Coordinated Development in Sino-foreign Cooperative Education among Universities of Beijing-Tianjin-Hebei Region (Research Project Number: HB21JY061).

## Conflict of interest

The authors declare that the research was conducted in the absence of any commercial or financial relationships that could be construed as a potential conflict of interest.

## Publisher's note

All claims expressed in this article are solely those of the authors and do not necessarily represent those of their affiliated organizations, or those of the publisher, the editors and the reviewers. Any product that may be evaluated in this article, or claim that may be made by its manufacturer, is not guaranteed or endorsed by the publisher.

## References

[1] Graham A Phelps R Maddison C Fitzgerald R. Supporting children's mental health in schools: teacher views. Teach Teach. (2011) 17:479–96. 10.1080/13540602.2011.580525

[2] Yoo SY Choi YJ Um YJ. Challenges and growth as a mental health professional from volunteering experiences in the community gambling awareness campaign. Int J Mental Health Promot. (2020) 22:83–91. 10.32604/IJMHP.2020.011299

[3] Rickwood D Thomas K Brown PM Prowse H. Mental illness education through stories of lived experience: validation review of the Donoharm framework. Int J Mental Health Promot. (2021) 23:455–69. 10.32604/IJMHP.2021.017613

[4] Avey JB Luthans F Smith RM Palmer NF. Impact of positive psychological capital on employee well-being over time. J Occup Health Psychol. (2010) 15:17. 10.1037/a0016998 20063956

[5] Shen S Wang S Ahmed MZ Hiramoni FA Sha J Yan X. Protective factors for loneliness among adolescents during covid-19: role of the interpersonal relationships and sibling status. Int J Mental Health Promot. (2021) 2021:499–511. 10.32604/IJMHP.2021.018661

[6] Park N. The role of subjective well-being in positive youth development. Ann Am Acad Pol Soc Sci. (2004) 591:25–39. 10.1177/0002716203260078

[7] Yang Y Li T He L. The Relationship among Chinese adolescents' parental involvement, core self-evaluation and school adaptation. Int J Mental Health Promot. (2021) 23:521–31. 10.32604/IJMHP.2021.019290

[8] Diener E Suh EM Lucas RE Smith HL. Subjective well-being: Three decades of progress. Psychol Bull. (1999) 125:276. 10.1037/0033-2909.125.2.276

[9] Henrich G Herschbach P. Questions on Life Satisfaction (FLZM): a short questionnaire for assessing subjective quality of life. Eur J Psychol Assessment. (2000) 16:150. 10.1027//1015-5759.16.3.150

[10] Shi C Yan J Wang L Shen H. Exploring the self-reported physical fitness and self-rated health, mental health disorders, and body satisfaction among Chinese adolescents: a cross-sectional study. Front Psychol. (2022) 2022:13. 10.3389/fpsyg.2022.1003231 36186394 PMC9521502

[11] Antaramian SP Huebner ES Hills KJ Valois RF. A dual-factor model of mental health: toward a more comprehensive understanding of youth functioning. Am J Orthopsychiatry. (2010) 80:462–72. 10.1111/j.1939-0025.2010.01049.x 20950287

[12] Strózik D Strózik T Szwarc K. The subjective well-being of school children. The first findings from the children's worlds study in Poland. Child Indic Res. (2016) 9:39–50. 10.1007/s12187-015-9312-8 26925176 PMC4757598

[13] Prasoon R Chaturvedi K. Life satisfaction: a literature review. Res Int J Manage Human Soc Sci. (2016) 1:24–31.

[14] Haranin EC Huebner ES Suldo SM. Predictive and incremental validity of global and domain-based adolescent life satisfaction reports. J Psychoeduc Assess. (2007) 25:127–38. 10.1177/0734282906295620

[15] Martin K Huebner ES Valois RF. Does life satisfaction predict victimization experiences in adolescence? Psychol Sch. (2008) 45:705–14. 10.1002/pits.2033634853828

[16] Herman KM Hopman WM Rosenberg MW. Self-rated health and life satisfaction among Canadian adults: associations of perceived weight status versus BMI. Qual Life Res. (2013) 22:2693–705. 10.1007/s11136-013-0394-9 23539466

[17] Suldo SM Huebner ES. Is extremely high life satisfaction during adolescence advantageous? Soc Indic Res. (2006) 78:179–203. 10.1007/s11205-005-8208-2

[18] Valois RF Zullig KJ Huebner ES Drane JW. Relationship between life satisfaction and violent behaviors among adolescents. Am J Health Behav. (2001) 25:353–66. 10.5993/AJHB.25.4.1 11488546

[19] Hawkins WE Hawkins MJ Seeley J. Stress, health-related behavior and quality of life on depressive symptomatology in a sample of adolescents. Psychol Rep. (1992) 71:183–6. 10.2466/pr0.1992.71.1.183 1529054

[20] Park HS Koo HY Schepp KG. Predictors of Suicidal Ideation for Adolescents by Gender. J Korean Acad Nurs. (2005) 35:1433. 10.4040/jkan.2005.35.8.1433 16415624

[21] Bisegger C Cloetta B Von Bisegger U Abel T Ravens-Sieberer U. Health-related quality of life: gender differences in childhood and adolescence. Sozial- und Präventivmedizin SPM. (2005) 50:281–91. 10.1007/s00038-005-4094-2 16300172

[22] Ravens-Sieberer U Görtler E Bullinger M. Subjective health and health behavior of children and adolescents–a survey of Hamburg students within the scope of school medical examination. Gesundheitswesen (Bundesverband der Arzte des Offentlichen Gesundheitsdienstes (Germany)). (2000) 62:148–55. 10.1055/s-2000-10487 10815341

[23] Kim J Byon KK. Leisure activities, happiness, life satisfaction, and health perception of older Korean Adults. Int J Mental Health Promot. (2021) 155–66. 10.32604/IJMHP.2021.01523228991205

[24] Yuan Z Xiang Y Chen Z. Mindfulness associates life satisfaction: the mediating role of internal control and the presence of meaning in life. Int J Mental Health Promot. (2021) 23:15–25. 10.32604/IJMHP.2021.012787

[25] Rodriguez-Ayllon M Cadenas-Sánchez C Estévez-López F Muñoz NE Mora-Gonzalez J Migueles JH. Role of physical activity and sedentary behavior in the mental health of preschoolers, children and adolescents: a systematic review and meta-analysis. Sports Med. (2019) 49:1383–410. 10.1007/s40279-019-01099-5 30993594

[26] Shen H Yan J Hong JT Clark C Yang XN Liu Y. Prevalence of physical activity and sedentary behavior among Chinese children and adolescents: variations, gaps, and recommendations. Int J Environ Res Public Health. (2020) 17:3066. 10.3390/ijerph17093066 32354193 PMC7246713

[27] Chen ST Yan J. Prevalence and selected sociodemographic of movement behaviors in schoolchildren from low-and middle-income families in Nanjing, China: a cross-sectional questionnaire survey. Children. (2020) 7:13. 10.3390/children7020013 32069924 PMC7072474

[28] Proctor CL Linley PA Maltby J. Youth Life Satisfaction: A Review of the Literature. J Happiness Stud. (2009) 10:583–630. 10.1007/s10902-008-9110-9

[29] Zullig KJ White RJ. Physical activity, life satisfaction, and self-rated health of middle school students. Appl Res Qual Life. (2011) 6:277–89. 10.1007/s11482-010-9129-z

[30] Valois RF Zullig KJ Huebner ES Drane JW. Physical activity behaviors and perceived life satisfaction among public high school adolescents. J School Health. (2004) 74:59–65. 10.1111/j.1746-1561.2004.tb04201.x 15077500

[31] Chen S Ho WKY Ahmed MD. Physical activity and its relationship with life satisfaction among middle school students: a cross-culture study. Sustainability. (2020) 12:6932. 10.3390/su12176932

[32] Ren T Yan J Sun Q. Sociodemographic Correlates of Organized Sports Participation in a Sample of Middle School Students in China. Front Public Health. (2021) 9:730555. 10.3389/fpubh.2021.730555 34869148 PMC8636987

[33] Matin N Kelishadi R Heshmat R Motamed-Gorji N Djalalinia S Motlagh ME. Joint association of screen time and physical activity on self-rated health and life satisfaction in children and adolescents: the CASPIAN-IV study. Int Health. (2017) 9:58–68. 10.1093/inthealth/ihw044 27836949

[34] Liu S Yu Q Hossain M Doig S Bao R Zhao Y. Meeting 24-h movement guidelines is related to better academic achievement: findings from the YRBS 2019 cycle. Int J Mental Health Promot. (2022) 24:13–24. 10.32604/IJMHP.2021.017660

[35] Pierannunzio D Spinelli A Berchialla P Borraccino A Charrier L Dalmasso P. Physical activity among Italian adolescents: association with life satisfaction, self-rated health and peer relationships. Int J Environ Res Public Health. (2022) 19:4799. 10.3390/ijerph19084799 35457667 PMC9032550

[36] Cantril H. The Pattern of Human Concern New Brunswick. NJ: Rutgers Univ Press (1965).

[37] Cheung F Lucas RE. Assessing the validity of single-item life satisfaction measures: Results from three large samples. Qual Life Res. (2014) 23:2809–18. 10.1007/s11136-014-0726-4 24890827 PMC4221492

[38] Jovanović V. The validity of the Satisfaction with Life Scale in adolescents and a comparison with single-item life satisfaction measures: a preliminary study. Qual Life Res. (2016) 25:3173–80. 10.1007/s11136-016-1331-5 27262574

[39] Joshanloo M Jovanović V. The relationship between gender and life satisfaction: analysis across demographic groups and global regions. Arch Women's Mental Health. (2020) 23:331–8. 10.1007/s00737-019-00998-w 31482245

[40] WHO Regional Office for Europe. Spotlight on Adolescent Health and Well-Being. Findings from the 2017/2018 Health Behaviour in School-Aged. WHO Regional Office for Europe: Copenhagen, Denmark. ISBN 978-92-890-5500-0 (2020).

[41] Mayers M. Combating *Pediatric Obesity with a Community-Based, Family-Centered Pediatric Obesity Prevention Program* (2020).

[42] Schenkel K. Health behavior in the context of digitalisation: challenges and opportunities of digital technologies. Zurich: University of Zurich (2021).

[43] Sanders TG. Investigating associations between neighbourhood green space and weight status: a longitudinal study of Australian children aged 4 to 13 years old. Australia: Western Sydney University (2016).

[44] Wilcox KR. Promoting Vegetable Consumption Among 4-and 5-Year-Old Students: Evaluation of a Multi-Component Nutrition Education Program (2011).

[45] Farren GL Zhang T Gu X Thomas KT. Sedentary behavior and physical activity predicting depressive symptoms in adolescents beyond attributes of health-related physical fitness. J Sport Health Sci. (2018) 7:489–96. 10.1016/j.jshs.2017.03.008 30450259 PMC6226422

[46] Bélair MA Kohen DE Kingsbury M Colman I. Relationship between leisure time physical activity, sedentary behaviour and symptoms of depression and anxiety: evidence from a population-based sample of Canadian adolescents. BMJ Open. (2018) 8:e021119. 10.1136/bmjopen-2017-021119 30337306 PMC6196847

[47] Costigan SA Lubans DR Lonsdale C Sanders T del Pozo Cruz B. Associations between physical activity intensity and well-being in adolescents. Prev Med. (2019) 125:55–61. 10.1016/j.ypmed.2019.05.009 31125627

[48] Oberste M Medele M Javelle F Lioba Wunram H Walter D Bloch W. Physical activity for the treatment of adolescent depression: a systematic review and meta-analysis. Front Physiol. (2020) 11:185. 10.3389/fphys.2020.00185 32265725 PMC7096373

[49] Eime RM Young JA Harvey JT Charity MJ Payne WR. A systematic review of the psychological and social benefits of participation in sport for children and adolescents: informing development of a conceptual model of health through sport. Int J Behav Nutr Phys Act. (2013) 10:1–21. 10.1186/1479-5868-10-98 23945179 PMC3751802

[50] Chmelík F Frömel K Groffik D Šafár M Mitáš J. Does vigorous physical activity contribute to adolescent life satisfaction? Int J Environ Res Public Health. (2021) 18:2236. 10.3390/ijerph18052236 33668269 PMC7956582

[51] Slapšinskaite A Lukoševičiute J Šmigelskas K. Interplay between adolescent physical activity and life satisfaction: gender as potential effect modifier. Int J Public Health. (2020) 65:1355–63. 10.1007/s00038-020-01473-5 32880659

[52] Kleszczewska D Dzielska A Salonna F Mazur J. The association between physical activity and general life satisfaction in lower secondary school students: the role of individual and family factors. Comm Ment Health J. (2018) 54:1245–52. 10.1007/s10597-018-0309-x 30076505 PMC6208981

[53] Landry BW Driscoll SW. Physical activity in children and adolescents. PM&R. (2012) 4:826–32. 10.1016/j.pmrj.2012.09.585 23174545

[54] Martha BA Vacchi CDO Fattori RA Macagnan FE. Effect of physical exercise on the functional capacity of children and adolescents submitted to transplantation of hematopoietic stem cells—a systematic review with meta-analysis. J Child Health Care. (2021) 25:18–30. 10.1177/1367493520903626 32013540

[55] Biddle SJH Ciaccioni S Thomas G Vergeer I. Physical activity and mental health in children and adolescents: An updated review of reviews and an analysis of causality. Psychol Sport Exerc. (2019) 42:146–55. 10.1016/j.psychsport.2018.08.011

[56] Garcia C Teles J Barrigas C Fragoso I. Health-related quality of life of Portuguese children and adolescents according to their biological maturation and volume of physical activity. Qual Life Res. (2018) 27:1483–92. 10.1007/s11136-018-1822-7 29502325

[57] Olofsson CK Skovdahl P Fridolfsson J Arvidsson D Börjesson M Sunnegårdh J. Life satisfaction, health-related quality of life and physical activity after treatment for valvular aortic stenosis. Cardiol Young. (2022) 30:1–7. 10.1017/S1047951122000920 35351216

[58] Cao H Qian Q Weng T Yuan C Sun Y Wang H. Screen time, physical activity and mental health among urban adolescents in China. Prevent Med. (2011) 53:316–20. 10.1016/j.ypmed.2011.09.002 21933680

[59] Urchaga JD Guevara RM Cabaco AS Moral-García JE. Life satisfaction, physical activity and quality of life associated with the health of school-age adolescents. Sustainability. (2020) 12:9486. 10.3390/su12229486

