# Supplementary material for: Association between vigorous physical activity and life satisfaction in adolescents
Source: Front Public Health. 2022 Oct 13;10:944620. doi: 10.3389/fpubh.2022.944620 (PMC9607945; doi:10.3389/fpubh.2022.944620)
Supplement: Supplementary file 1 [file Table_1.docx]

Supplementary Table S1. Sample characteristics

|  |  | N/Mean | %/SD |
| --- | --- | --- | --- |
| Gender |  |  |  |
|  | Boy | 105414 | 49.2 |
|  | Girl | 108666 | 50.8 |
| Age category |  |  |  |
|  | 11 | 68415 | 32.2 |
|  | 13 | 73472 | 34.6 |
|  | 15 | 70339 | 33.1 |
| Alcohol use last 30 days |  |  |  |
|  | Never | 161797 | 80.3 |
|  | 1-2 days | 24341 | 12.1 |
|  | 3-5 days | 7949 | 3.9 |
|  | 6-9 days | 3705 | 1.8 |
|  | 10-19 days | 1817 | 0.9 |
|  | 20-29 days | 479 | 0.2 |
|  | 30 days (or more) | 1512 | 0.8 |
| Family well off |  |  |  |
|  | Very well off | 39777 | 19.4 |
|  | Quite well off | 70361 | 34.4 |
|  | Average | 80002 | 39.1 |
|  | Not very well off | 11665 | 5.7 |
|  | Not at all well off | 2983 | 1.5 |
| Smoking |  |  |  |
|  | Every day | 7386 | 3.5 |
|  | Once a week | 3814 | 1.8 |
|  | Less than once a week | 5585 | 2.7 |
|  | Don't | 193610 | 92.0 |
| Watch tv/dvd/video, weekdays |  |  |  |
|  | None at all | 10508 | 5.5 |
|  | Half an hour a day | 27861 | 14.5 |
|  | 1 hour a day | 41730 | 21.7 |
|  | 2 hours a day | 45455 | 23.7 |
|  | 3 hours a day | 29939 | 15.6 |
|  | 4 hours a day | 16323 | 8.5 |
|  | 5 hours a day | 8657 | 4.5 |
|  | 6 hours a day | 3620 | 1.9 |
|  | 7 hours or more a day | 7888 | 4.1 |
| Watch tv/dvd/video, weekends |  |  |  |
|  | None at all | 6877 | 3.7 |
|  | Half an hour a day | 13851 | 7.5 |
|  | 1 hour a day | 24422 | 13.2 |
|  | 2 hours a day | 36816 | 19.9 |
|  | 3 hours a day | 33507 | 18.1 |
|  | 4 hours a day | 25962 | 14.0 |
|  | 5 hours a day | 16886 | 9.1 |
|  | 6 hours a day | 9728 | 5.3 |
|  | 7 hours or more a day | 17040 | 9.2 |
| Play computer games, weekdays |  |  |  |
|  | None at all | 36511 | 19.0 |
|  | Half an hour a day | 39122 | 20.4 |
|  | 1 hour a day | 37164 | 19.3 |
|  | 2 hours a day | 29973 | 15.6 |
|  | 3 hours a day | 18664 | 9.7 |
|  | 4 hours a day | 11162 | 5.8 |
|  | 5 hours a day | 6990 | 3.6 |
|  | 6 hours a day | 3489 | 1.8 |
|  | 7 hours or more a day | 9135 | 4.8 |
| Play computer games, weekends |  |  |  |
|  | None at all | 25392 | 13.7 |
|  | Half an hour a day | 26927 | 14.5 |
|  | 1 hour a day | 28759 | 15.5 |
|  | 2 hours a day | 28445 | 15.4 |
|  | 3 hours a day | 21774 | 11.8 |
|  | 4 hours a day | 16597 | 9.0 |
|  | 5 hours a day | 11737 | 6.3 |
|  | 6 hours a day | 7830 | 4.2 |
|  | 7 hours or more a day | 17642 | 9.5 |
| Computer use, weekdays |  |  |  |
|  | None at all | 19135 | 10.0 |
|  | Half an hour a day | 36928 | 19.3 |
|  | 1 hour a day | 39339 | 20.5 |
|  | 2 hours a day | 32306 | 16.8 |
|  | 3 hours a day | 21326 | 11.1 |
|  | 4 hours a day | 13678 | 7.1 |
|  | 5 hours a day | 9288 | 4.8 |
|  | 6 hours a day | 5302 | 2.8 |
|  | 7 hours or more a day | 14444 | 7.5 |
| Computer use, weekends |  |  |  |
|  | None at all | 17760 | 9.6 |
|  | Half an hour a day | 26486 | 14.3 |
|  | 1 hour a day | 30211 | 16.4 |
|  | 2 hours a day | 29197 | 15.8 |
|  | 3 hours a day | 22449 | 12.2 |
|  | 4 hours a day | 16755 | 9.1 |
|  | 5 hours a day | 12205 | 6.6 |
|  | 6 hours a day | 8537 | 4.6 |
|  | 7 hours or more a day | 21077 | 11.4 |
| Vigorous physical activity, frequency |  |  |  |
|  | Every day | 38085 | 19.2 |
|  | 4-6 times a week | 48348 | 24.4 |
|  | 2-3 times a week | 57879 | 29.2 |
|  | Once a week | 25958 | 13.1 |
|  | Once a month | 7438 | 3.8 |
|  | Less than once a month | 9289 | 4.7 |
|  | Never | 11114 | 5.6 |
| Exercise - hours a week |  |  |  |
|  | None | 20737 | 10.2 |
|  | Half an hour | 31296 | 15.4 |
|  | 1 hour | 45887 | 22.6 |
|  | 2-3 hours | 52780 | 26.0 |
|  | 4-6 hours | 29748 | 14.7 |
|  | 7 hours or more | 22342 | 11.0 |
| Body Mass Index |  | 19.6 | 3.5 |
| Life satisfaction |  | 7.6 | 1.9 |
